# Bananas as an Energy Source during Exercise: A Metabolomics Approach

**DOI:** 10.1371/journal.pone.0037479

**Published:** 2012-05-17

**Authors:** David C. Nieman, Nicholas D. Gillitt, Dru A. Henson, Wei Sha, R. Andrew Shanely, Amy M. Knab, Lynn Cialdella-Kam, Fuxia Jin

**Affiliations:** 1 Human Performance Laboratory, Appalachian State University, North Carolina Research Campus, Kannapolis, North Carolina, United States of America; 2 Dole Nutrition Research Laboratory, North Carolina Research Campus, Kannapolis, North Carolina, United States of America; 3 Department of Biology, Appalachian State University, Boone, North Carolina, United States of America; 4 Bioinformatics Services Division, University of North Carolina at Charlotte, North Carolina Research Campus, Kannapolis, North Carolina, United States of America; Universidad Europea de Madrid, Spain

## Abstract

This study compared the acute effect of ingesting bananas (BAN) versus a 6% carbohydrate drink (CHO) on 75-km cycling performance and post-exercise inflammation, oxidative stress, and innate immune function using traditional and metabolomics-based profiling. Trained cyclists (N = 14) completed two 75-km cycling time trials (randomized, crossover) while ingesting BAN or CHO (0.2 g/kg carbohydrate every 15 min). Pre-, post-, and 1-h-post-exercise blood samples were analyzed for glucose, granulocyte (GR) and monocyte (MO) phagocytosis (PHAG) and oxidative burst activity, nine cytokines, F_2_-isoprostanes, ferric reducing ability of plasma (FRAP), and metabolic profiles using gas chromatography-mass spectrometry. Blood glucose levels and performance did not differ between BAN and CHO (2.41±0.22, 2.36±0.19 h, P = 0.258). F_2_-isoprostanes, FRAP, IL-10, IL-2, IL-6, IL-8, TNFα, GR-PHAG, and MO-PHAG increased with exercise, with no trial differences except for higher levels during BAN for IL-10, IL-8, and FRAP (interaction effects, P = 0.003, 0.004, and 0.012). Of 103 metabolites detected, 56 had exercise time effects, and only one (dopamine) had a pattern of change that differed between BAN and CHO. Plots from the PLS-DA model visualized a distinct separation in global metabolic scores between time points [R^2^Y(cum) = 0.869, Q^2^(cum) = 0.766]. Of the top 15 metabolites, five were related to liver glutathione production, eight to carbohydrate, lipid, and amino acid metabolism, and two were tricarboxylic acid cycle intermediates. BAN and CHO ingestion during 75-km cycling resulted in similar performance, blood glucose, inflammation, oxidative stress, and innate immune levels. Aside from higher dopamine in BAN, shifts in metabolites following BAN and CHO 75-km cycling time trials indicated a similar pattern of heightened production of glutathione and utilization of fuel substrates in several pathways.

## Introduction

Heavy exertion induces transient inflammation and oxidative stress, and wide ranging perturbations in the immune system [1]–[7]. Various nutritional agents have been tested for their capacity to attenuate these indicators of physiologic stress while supporting exercise performance and recovery [2].

Bananas are a cost effective energy source and used by endurance athletes because of the perception that they are a good source of carbohydrate and potassium. One medium banana (∼118 g) contains about 27 g carbohydrate (half as sugars), 3.1 g dietary fiber, 105 kilocalories, and is a good source of potassium (422 mg) and vitamin B6 (0.43 mg) [8]. The 14.4 g sugars in a medium banana are a mixture of glucose (5.9 g), fructose (5.7 g), and sucrose (2.8 g) [8]. The glycemic index of bananas is 51 (low-to-medium rating), similar to grapes, mangos, pineapples, raisins, macaroni, orange juice, and honey [9]. The antioxidant value of bananas described in ORAC units is 1,037 µmol TE, which is similar to kiwi fruit and orange juice [10]. Thus bananas appear to be a unique mixture of carbohydrates, nutrients, and antioxidants that may provide good nutrition support during prolonged and intensive exercise, but published data from studies with human athletes are lacking [11].

In previous studies conducted by our research group, we showed that 60 g carbohydrate per hour in beverage form relative to placebo partially countered exercise-induced increases in cytokines and changes in innate immunity [3]–[5]. The purpose of this study was to compare the acute effect of ingesting bananas versus a 6% carbohydrate beverage on 75-km cycling time trial performance, exercise-induced inflammation, oxidative stress and capacity, and changes in immune function in trained cyclists. Metabolomics is the measurement of small molecules or metabolites present in biological samples to elucidate the effect of a particular stimulus on metabolic pathways, and is being increasingly used in sports nutrition research [2]–[18]. The complex relationships between the use of whole foods or nutrient cocktails by athletes during exercise are best explored using the tool of metabolomics. To improve interpretation of underlying metabolic processes in the comparison between bananas and the 6% carbohydrate beverage, pre- and post-exercise blood samples were analyzed for non-targeted shifts in metabolites using gas chromatography mass spectrometry.

## Methods

### Subjects

Subjects included 14 male cyclists (ages 18–45) who regularly competed in road races (category 1 to 5) and had experience with cycling time trials. Subjects trained normally, maintained weight, and avoided the use of large-dose vitamin and mineral supplements, herbs, and medications known to affect inflammation and immune function for the duration of the study. All subjects signed informed consent and all study procedures were approved by the Institutional Review Board at Appalachian State University.

### Research Design

One week prior to the first 75-km time trial, each athlete completed orientation/baseline testing in the North Carolina Research Campus Human Performance Laboratory operated by Appalachian State University in Kannapolis, NC. Demographic and training histories were acquired with questionnaires. During orientation, a dietitian instructed the subjects to follow a diet moderate in carbohydrate (using a provided food list) during the 3-d period before each 75-km time trial. Subjects recorded food intake in 3-d food records, and were then analyzed using a computerized dietary assessment program for energy and macronutrient content (Food Processor; ESHA Research, Salem, OR).

During baseline testing, maximal power, oxygen consumption, ventilation, and heart rate were measured during a graded exercise test (25 Watts increase every two minutes, starting at 150 Watts) with the Cosmed Quark CPET metabolic cart (Rome, Italy) and the Lode cycle ergometer (Lode Excaliber Sport, Lode B.V., Groningen, Netherlands). Body composition was measured with the Bod Pod body composition analyzer (Life Measurement, Concord, CA).

One week following baseline testing, subjects completed the first 75-km time trial. Subjects were randomized to banana and 6% carbohydrate beverage conditions, and then crossed over to the opposite condition during the second 75-km time trial three weeks later. On the date of each 75-km time trial session, subjects consumed a standardized meal at 12:00 noon consisting of Boost Plus at 10 kcal/kg (41.9 kJ/kg) (Boost Plus; Mead Johnson Nutritionals, Evansville, IN). Subjects reported to the lab at 2:45 pm and provided a blood sample. At 2:50 pm, subjects ingested 0.4 g/kg carbohydrate from bananas (BAN) or from a standard 6% carbohydrate beverage (CHO) (Gatorade™, Chicago, IL). Subjects ingested 0.2 g/kg body weight every 15 minutes of BAN or CHO during the 75-km time trials. BAN were consumed with water to equal what was consumed with CHO. BAN were provided by Dole Foods (Westlake Village, CA) and were at a level six ripening stage (completely yellow with no brown spots).

Subjects cycled (3:00 pm start) on their own bicycles on CompuTrainer Pro Model 8001 trainers (RacerMate, Seattle, WA) with heart rate and rating of perceived exertion (RPE) recorded every 30 minutes, and workload continuously monitored using the CompuTrainer MultiRider software system (version 3.0, RacerMate, Seattle, WA). A mountainous 75-km course with moderate difficulty was chosen and programmed into the software system for use in each time trial. Fingertip capillary blood samples were drawn using heparin-lined microcapillary tubes pre-exercise, 1-h into the 75-km time trial, and post-exercise. Blood samples were immediately placed in microfuge tubes lined with EDTA dipotassium salt (RAM Scientific Inc., Germany), and analyzed using the YSI 2300 STAT Plus Glucose and Lactate analyzer (Yellow Springs, OH).

Blood samples were taken via venipuncture immediately after completing the 75-km time trial, and then 1-hr post-exercise. Subjects completed symptom logs, which included questions on digestive health (heartburn, bloating, diarrhea, and nausea). Subjects indicated responses using a 12-point Likert scale, with 1 relating to “none at all”, 6 “moderate”, and 12 “very high”.

### Complete Blood Count

Routine complete blood counts were performed by our clinical hematology laboratory using a Coulter Ac.TTM 5Diff Hematology Analyzer (Beckman Coulter, Inc., Miami, FL) and provided hemoglobin and hematocrit for the determination of plasma volume change [19].

### Plasma Cytokines

Total plasma concentrations of nine inflammatory cytokines (IL-6, TNFα, granulocyte-macrophage colony stimulating factor [GM-CSF], IFNγ, IL-1β, IL-2, IL-8, IL-10, and IL-12p70) were determined using an electrochemiluminescence based solid-phase sandwich immunoassay (Meso Scale Discovery, Gaithersburg, MD, USA). All samples and provided standards were analyzed in duplicate, and the intra-assay CV ranged from 1.7 to 7.5% and the inter-assay CV 2.4 to 9.6% for all cytokines measured. The minimum detectable concentration of IL-6 was 0.27 pg/ml, TNFα 0.50 pg/ml, GM-CSF 0.20 pg/ml, IFNγ 0.53 pg/ml, IL-1β 0.36 pg/ml, IL-2 0.35 pg/ml, IL-8 0.09 pg/ml, IL-10 0.21 pg/ml, and IL-12p70 1.4 pg/ml. Pre- and post-exercise samples for the cytokines were analyzed on the same assay plate to decrease inter-kit assay variability.

### Oxidative Stress And Antioxidant Capacity

Plasma F_2_-isoprostanes were determined using gas chromatography mass spectrometry (GC-MS) [20]. Plasma was collected from heparinized blood, immediately flash-frozen in liquid nitrogen, and stored at −80°C. Immediately prior to assay plasma samples were thawed. The samples were used to extract free F_2_-isoprostanes with deuterated [^2^H_4_] prostaglandin F_2α_ (PGF_2α_) added as an “internal” standard. The mixture was then added to a C18 Sep Pak column, followed by silica solid phase extractions. F_2_-isoprostanes were converted to pentafluorobenzyl esters, subjected to thin layer chromatography, and converted to trimethylsilyl ether derivatives. Samples were analyzed by a negative ion chemical ionization GC-MS using an Agilent 6890N gas chromatography interfaced to an Agilent 5975B inert MSD mass spectrometer (Agilent Technologies, Inc. Santa Clara, CA).

Total plasma antioxidant power was determined by the ferric reducing ability of plasma (FRAP) assay, a single electron transfer reaction [21]. This assay utilizes water soluble antioxidants native to the plasma collected from EDTA treated blood to reduce ferric iron to the ferrous form subsequently producing a chromogen identifiable at 593 nm. Samples and standards are expressed as ascorbate equivalents based on an ascorbate standard curve. Intra-assay and inter-assay coefficients of variation were less than 5% and 7%, respectively.

### Analysis of Dopamine in Cavendish Bananas

Dopamine hydrochloride (4-(2-aminoethyl)benzene-1,2-diol hydrochloride, 99%) was purchased from Acros Organics (New Jersey). Cavendish bananas (ripening stage 6) were obtained from a local grocery store and assayed on the day of purchase. Approximately 50 grams of banana flesh were blended with 150 mls of 70% aqueous methanol for three minutes, and analyzed for dopamine content using liquid chromatography electrospray ionization tandem mass spectrometry (LC-ESI-MS-MS) (Thermo Scientific LTQ Velos system, West Palm Beach, FL).

### Granulocyte and Monocyte Phagocytosis, Oxidative Burst Activity

Phagocytosis was measured through the uptake of FITC-labeled bacteria and oxidative burst was measured through the oxidation of non-fluorescent hydroethidine (HE) to fluorescent ethidium bromide in cells stimulated with unlabeled bacteria. Unlabeled and FITC-labeled bacteria (Staphylococcus aureus; Molecular Probes, Eugene, OR) were suspended in phosphate buffered saline (PBS) to working concentrations of 133,333 particles/µL. For each sample, 100 µL of blood were dispensed into two polypropylene tubes. To one tube, 10 µL of HE working solution (10 µg HE/mL; Molecular Probes) were added and the tubes incubated at 37°C for 15 min, then cooled at 4°C for 12 min. Using a bacteria to phagocyte (neutrophils and monocytes) ratio of 8∶1, unlabeled bacteria were added to the HE loaded tubes and FITC-labeled bacteria were added to the second tube in the set. Both tubes were incubated at 37°C for 20 min, placed in an ice water bath and 100 µL of quench solution were added to allow suppression of surface bound FITC-bacteria fluorescence. Cells were washed twice with cold PBS and resuspended in 50 µL cold fetal bovine serum. Samples were processed on a Q-Prep™ Workstation (Beckman Coulter, Inc) and analysis was performed within 18-hr of blood collection using a Beckman Coulter FC-500 flow cytometer. After gating on the granulocyte and monocyte populations using forward scatter and side scatter, the mean fluorescence intensity (MFI; x-mean) and percent positive cells for FITC (FL_1_) and oxidized HE (FL_2_) were determined.

### Metabolomics

All samples (both plasma extracts and standards for the internal library) were analyzed on an Agilent 7890A GC system coupled to an Agilent 5975C EI/CI Mass Selective Detector. The raw data files generated by GC-MS were converted to NetCDF format. The converted data were processed using Leco ChromaTOF software v4.24 (St. Joseph, MI) including baseline de-nosing, smoothing, peak picking, and peak signal alignment (signal-to-noise ≥30). Metabolite annotation was performed by comparing unknown signal patterns from the study samples to those of reference standards from an internal library containing approximately 600 human metabolites (Sigma-Aldrich, St. Louis, MO) established on the GC-MS system. Commercial libraries including the NIST library 2008 and LECO/Fiehn Metabolomics Library for GC-MS metabolome data (similarity threshold of 70%) were also used for additional compound annotation. Heptadecanoic acid was added to the study samples as an internal standard to monitor analytical variations during the entire sample preparation and analysis processes, and precision was calculated by injecting six randomly selected samples five times. The average CV for heptadecanoic acid was less than 5%, and the mean CV across the entire sample analysis was 15.3%.

### Statistical Analysis

All data are expressed as mean ± SD. The biomarker data were analyzed using a 2 (condition)×3 (time) repeated-measures ANOVA, within-subject design. When interaction effects were significant (P≤0.05), changes between time points within BAN or CHO conditions were compared between trials using paired t tests, with significance set after Bonferroni adjustment at P≤0.025. For metabolomics data, a linear model with repeated measures was used to examine the effect of treatment (BAN or CHO) and time (pre-exercise, immediate post-exercise, 1 hour post-exercise) on metabolite concentration, where metabolite concentration was the response variable, and treatment, time, and treatment×time interaction were predictor variables. Due to the crossover design, the sequence (BAN→CHO or CHO→BAN), and visit (first or second) effects were also adjusted in the model. This analysis was performed in the MIXED procedure in SAS (version 9.2, SAS Institute, Inc., Cary, NC), and was performed for each metabolite separately. Benjamini-Hochberg method for False Discovery Rate (FDR) correction in the MULTTEST procedure in SAS was used for multiple testing correction. To improve the normality of the data, the concentration of each metabolite was log transformed, and outliers with studentized residue >3 or <−3 were excluded. Metabolites with significant (FDR adjusted p-value<0.05) treatment×time interaction effect were considered to have significantly different responses to the two treatments. Metabolites with significant (FDR adjusted p-value<0.05) time effect were considered to be significantly affected by exercise within a carbohydrate-fed context. Missing values for a given metabolite were imputed with the observed minimum after the normalization step. Partial Least Square Discriminant Analysis (PLS-DA) in SIMCA-P+ (Version 12, Umetrics, Umeå, Sweden) was used to detect metabolites that best distinguished the three time points. The default 7-round cross-validation in the SIMCA-P software package was applied with 1/7 of the samples being left out from the mathematical model in each round. Variable Influence on Projection (VIP) score was calculated based on the PLS weights and the variability explained in PLS-DA. Metabolites with VIP>1 were considered the most important metabolites responsible for the differentiation of the three time points. Similarly, PLS-DA was used to detect metabolites that best distinguished BAN from CHO treatment. In this analysis, the ratio of immediate post-exercise/pre-exercise for each subject was calculated, and used as input data for PLS-DA.

## Results

Fourteen subjects completed all aspects of the study, and subject characteristics indicated that they were well trained and experienced cyclists (mean age 37.0±7.1 y, body fat 17.8±4.5%, maximal power 379±46.8 Watts, VO_2max_ 58.6±5.2 ml^.^kg^.−1^min^−1^, training and racing history 8.4±6.4 y). Subjects averaged 272±86.1 km/wk during the 3-month period prior to the study. Three-day food records before each of the two time trials revealed no significant differences in energy or macronutrient intake. Energy intake was 2486±625 kcal/day (10.5±2.47 MJ/day) and 2539±662 kcal/day (10.2±2.66 MJ/day), with carbohydrate representing 60.4±5.6% and 59.4±6.0%, protein 15.9±2.2% and 16.1±3.4%, and fat 23.7±5.6% and 24.5±5.2% of total energy for BAN and CHO conditions, respectively. The three-day food records also revealed no significant differences in potassium 2041±700 mg and 2454±625 mg, vitamin C 102±58.0 and 115±76.0 mg, and fiber 30.5±10.3 g and 33.8±11.0 g intake for BAN and CHO, respectively.

Mean power (225±43.0, 233±43.8 Watts, P = 0.178), heart rate (91.1±4.9, 89.3±3.4%HR_max_, P = 0.096), rating of perceived exertion (14.6±1.5, 14.4±1.1 RPE units, P = 0.613), and total time (2.41±0.22, 2.36±0.19 h, P = 0.258) did not differ between BAN and CHO 75-km cycling time trials, respectively. The patterns of increase over time during the 75-km cycling trials were similar between BAN and CHO for serum glucose (23% and 19%, respectively, interaction effect, P = 0.849) and blood lactate (220% and 227%, respectively, interaction effect, P = 0.439). Mean carbohydrate intake during BAN and CHO trials was 150±19.5 grams. Subjects reported feeling significantly more full (P = 0.003) and bloated (P = 0.014) during the BAN versus CHO trial. Subjects lost 0.4 kg more body weight during the BAN versus CHO trial (mean weight change, −1.5±0.7, −1.1±1.1 kg, respectively, P = 0.015). Plasma volume shifts were less than 2% following exercise and did not differ between trials (P = 0.711).

The patterns of increase in plasma F_2_-isoprostanes did not differ between BAN and CHO trials (Table 1). The pattern of increase in FRAP pre-to-post exercise was higher in BAN compared to CHO (31% versus 18%, respectively, interaction effect, P = 0.012) (Table 1). Exercise-induced increases were measured for five of nine cytokines, with significantly higher post-75 km cycling levels in BAN for IL-8 and IL-10 (Table 1).

**Table 1:** Plasma cytokine, granulocyte (GR) and monocyte (MO) phagocytosis (PHAG) and oxidative burst activity (OBA) (mean fluorescence intensity or MFI), and oxidative stress measures pre-exercise, immediately post-exercise (75-km), and 1-hr post-exercise in cyclists under banana (BAN) and 6% carbohydrate beverage (CHO) conditions (mean±SD).

Variable	Pre-Exercise	Post-Exercise	1-h Post-Exercise	Time; interaction P values
**TNFα (pg/ml)**				
BAN	6.03±1.68	8.82±2.02	8.70±2.17	<0.001; 0.104
CHO	5.64±1.31	7.21±2.02	7.00±1.72	
**IL-6 (pg/ml)**				
BAN	0.90±0.56	14.6±9.35	10.9±7.93	<0.001; 0.540
CHO	0.99±0.56	12.2±9.80	8.66±7.86	
**IL-2 (pg/ml)**				
BAN	1.27±0.71	1.71±0.71	1.62±0.90	0.036; 0.185
CHO	1.50±0.86	1.52±0.71	1.48±0.79	
**IL-8 (pg/ml)**				
BAN	2.12±0.56	11.1±5.84*	8.82±3.14*	<0.001; 0.004
CHO	2.19±0.79	7.38±3.74	6.75±3.07	
**IL-10 (pg/ml)**				
BAN	2.61±1.57	11.0±5.13*	13.2±12.3*	0.003; 0.003
CHO	2.24±1.76	5.98±3.52	6.96±7.82	
**GR-PHAG (MFI)**				
BAN	270±71.1	373±134	435±171	0.001; 0.215
CHO	284±134	321±164	346±147	
**MO-PHAG (MFI)**				
BAN	137±50.9	233±104	281±117	<0.001; 0.191
CHO	156±87.6	206±117	224±100	
**GR-OBA (MFI)**				
BAN	12.0±4.94	12.2±4.12	11.6±4.60	0.492; 0.697
CHO	12.9±7.11	11.4±5.91	11.0±6.73	
**MO-OBA (MFI)**				
BAN	8.70±2.17	10.2±2.39	9.19±3.11	0.207; 0.810
CHO	8.95±3.89	9.56±3.67	9.13±5.05	
**F_2_-Isoprostanes (pg/ml)**				
BAN	80.2±14.4	104±15.5	98.5±13.4	<0.001; 0.145
CHO	82.9±11.9	98.3±15.3	90.3±9.95	
**FRAP (ascorbic acid equivalents, µmol/L)**				
BAN	460±51.3	604±79.3*	575±76.0*	<0.001; 0.012
CHO	442±71.8	521±75.6	510±63.6	

* P<0.025, difference in change, pre-exercise when comparing BAN and CHO conditions.

The pattern of increase in granulocyte (GR) and monocyte (MO) phagocytosis (PHAG) did not differ between BAN and CHO (Table 1). GR and MO oxidative burst activity (OBA) did not change post-exercise, and no trial differences were measured.

**Figure 1:**
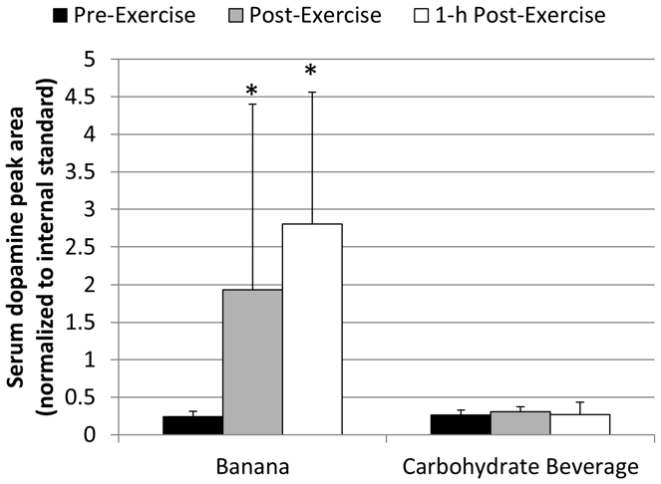
Serum dopamine peak area (normalized to internal standard) was higher in BAN compared to CHO following 75-km cycling (interaction effect, P<0.001). * P<0.025, difference in change from pre-exercise when comparing BAN and CHO. Data shown as mean±SD.

**Figure 2:**
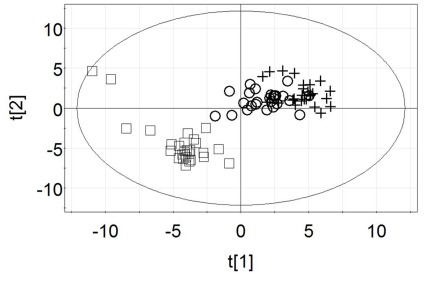
Score plots from the PLS-DA model visualize the global metabolic differences between pre-exercise (□), immediately post-exercise (+), and 1-h post-exercise (◯) for the combined data from the BAN and CHO beverage trials (75-km cycling). A satisfactory separation was obtained between time points groups (Q^2^Y = 0.766).

Of 103 metabolites detected through our GC-MS metabolomics system, 56 had significant time effects following the 75-km cycling bouts. Only one (dopamine) of the 56 metabolites had a pattern of change that differed between BAN and CHO, and overall treatment effects were not separated by PLS-DA modeling. Dopamine significantly increased in BAN compared to CHO, as shown in Figure 1 (interaction effect, P<0.001). Score plots from the PLS-DA model in Figure 2 visualized the global metabolic differences between pre-exercise, immediately post-exercise, and 1-h post-exercise, indicating a distinct separation between time points with some overlap between the two-post-exercise time points [R^2^Y(cum) = 0.869, Q^2^(cum) = 0.766]. Of the 56 metabolites with significant (FDR adjusted p-value<0.05) time and exercise effects, 25 had VIP scores greater than 1. Fold change (immediate post-exercise/pre-exercise) values for the top 15 metabolites (ranked by FDR adjusted p-values) are listed in Table 2. Of the 15 metabolites, five (2-hydroxybutyric acid, 2-aminobutyric acid, L-glutamic acid, L-methionine, and L-proline) were related to liver glutathione production, four (palmitoleic acid, palmitic acid, oleic acid, and heptadecanoic acid) to lipid metabolism, three (2,3,4-trihydroxybutanoic acid, D-fructose, and pyruvic acid) to carbohydrate metabolism, two (malic acid and succinic acid) were intermediate in the tricarboxylic acid cycle (TCA cycle), and one (L-isoleucine) was a branched chain amino acid.

**Table 2:** Fold change values for 15 metabolites changed immediately after the 75-km cycling time trials (VIP score >1.0 and ranked by FDR adjusted p-values, all <0.001).

Serum Metabolite	Mean Fold Change
Palmitoleic Acid	22.0
2,3,4-Trihydroxybutanoic Acid	3.1
Malic Acid	5.6
Succinic Acid	12.7
Palmitic Acid	3.9
Oleic Acid	6.1
Heptadecanoic Acid	2.8
D-Fructose	11.5
2-Hydroxybutyric Acid	1.9
L-Isoleucine	0.7
L-Glutamic Acid	1.7
2-Aminobutyric Acid	0.7
L-Methionine	0.9
Pyruvic Acid	2.8
L-Proline	0.9

LC-ESI-MS-MS analysis of banana flesh indicated a dopamine concentration of 0.42 mg/100 grams. The average subject ingested 794±94.3 grams banana during the banana cycling trial (6–7 total bananas), for a total banana dopamine intake of 3.33±0.41 mg.

## Discussion

These data from a randomized, crossover study with 14 trained cyclists indicate that acute ingestion of BAN or CHO supported 75-km cycling performance and underlying metabolic processes to a similar degree when the rate of carbohydrate delivery was equated. Exercise-induced inflammation, oxidative stress, and changes in innate immune function were also comparable between BAN and CHO trials, with the exception of a few biomarkers including IL-10 and IL-8. BAN compared to CHO resulted in higher antioxidant capacity (as measured through FRAP) and serum dopamine levels. The 75-km cycling time trials caused wide ranging increases in serum metabolites, and the data support a similar pattern of intensified production of glutathione and utilization of fuel substrates in several pathways during both BAN and CHO.

Metabolomics utilizes analytical technologies such as nuclear magnetic resonance (NMR) spectroscopy or mass spectrometry to measure the multicomponent metabolic composition of biological fluids such as urine, plasma, or serum [12], [22]. The use of metabolomics in disease states and pharmacological interventions is widespread, but applications to nutritional and exercise interventions have only recently begun [13]–[18], [22]. Comparisons between studies including metabolomics are dependent on multiple factors including the analytical technology and metabolite standards utilized, acute versus chronic context of the nutrition and exercise interventions, exercise workload, the use of urine versus serum or plasma, and the timing of collection of biological fluids during the intervention.

Using an untargeted approach with GC-MS analytical technology, our data indicate substantial shifts in metabolites related to glutathione production and fuel substrate usage following prolonged and intense exercise, with no differences between BAN and CHO. Our metabolomics data also support that these shifts in metabolites persisted, but with some attenuation, for at least one hour post-exercise. Lipid peroxidation increases during intense, prolonged exertion, as supported by the 20–30% increase in plasma F2-isoprostanes experienced by our subjects [7]. Glutathione is one of several important antioxidant defense systems, and the increase in five metabolites (2-hydroxybutyric acid, 2-aminobutyric acid, L-glutamic acid, L-methionine, and L-proline) is supportive of enhanced glutathione production [23]. We are unaware of other metabolomics-based exercise studies that report similar findings.

Lewis et al. [15] acquired plasma metabolic profiles using liquid chromatography mass spectrometry (LC-MS) in 25 runners who competed in the Boston Marathon (average time, 247±46 minutes). Despite the differences in MS platforms, many of the reported shifts in metabolites were similar to ours including increases in TCA cycle intermediates, and indicators of amino acid catabolism and increased glycolysis and lipolysis. Lehmann et al. [14] collected plasma samples from subjects running moderately either one hour or two hours, and using a non-targeted- metabolomics approach with LC-MS technology, reported that the most discriminant metabolite changes were medium and long chain acylcarnitines. This research group also showed in cell culture studies that acylcarnitines were released as intermediates of partial β-oxidation, and that octanoyl-, decanoyl-, and dodecanoylcarnitine were able to support the oxidation of palmitate, proving more effective than L-carnitine. Our GCMS platform standards library did not contain any acylcarnitine standards and therefore we were unable to confirm their presence in our samples. However, the largest fold shift of a metabolite experienced by our subjects was for palmitoleic acid (even under carbohydrate-fed conditions), a common fuel substrate found in human adipose tissue [24].

Dopamine is a polyphenolic found in small quantities in banana flesh, and in large quantities in the banana peel [25]. Acute ingestion of bananas (∼6–7 total) within a 2–3 h period resulted in a mean intake of 3.33 mg dopamine and a significant increase in serum free dopamine in our subjects, a finding with some published support [26]. After ingestion, an undetermined proportion of food dopamine is absorbed, with most rapidly conjugated by sulfate or glucuronide, blunting most of dopamine's biological activity [26]–[28]. The free form of dopamine constitutes less than 2% of total plasma dopamine [29]. Dopamine infusion at clinical levels results in an increase in blood pressure, pulse rate, and cardiac output, but the relatively small increase in dopamine sulfate or glucuronide after ingestion is not associated with a cardiovascular response [27], [30]. Dopamine is a strong water-soluble antioxidant, and together with other banana polyphenolics such as (+)-catechin (flavan-3-ols) may have contributed to the post-exercise increase in FRAP experienced by our subjects during the BAN trial [25], [31]. However, the improvement in antioxidant capacity associated with BAN did not result in lowered oxidative stress as measured with F2-isoprostanes when compared to CHO. Dopamine cannot cross the blood-brain barrier [29], and thus does not contribute to mood improvement during exercise, as evidenced by no differences in the rating of perceived exertion (RPE) between BAN and CHO trials in our subjects.

The CHO used in this study is sweetened with a sucrose-dextrose-fructose blend (each 0.5 liter contains 26.2 g total sugar, with 11.0 g glucose, 9.1 g fructose, and 4.6 g sucrose) [8]. One medium banana has 27.0 g carbohydrate, with 6.4 g starch, 5.9 g glucose, 5.7 g fructose, 2.8 g sucrose, and 3.1 g dietary fiber [8]. Despite dissimilar carbohydrate profiles, 75-km cycling performance and metabolic outcomes were similar when comparing CHO and BAN under conditions where total carbohydrate intake was matched. Subjects felt somewhat more full and bloated after cycling 75-km with BAN compared to CHO, and was probably related to the nearly 15 g dietary fiber consumed [8].

Mitchell et al. [11] reported no difference in 10-km treadmill runs when trained runners consumed similar amounts of carbohydrate from bananas and various types of beverages. The use of other whole foods such as raisins during exercise has been reported to support performance and blood glucose levels to a comparable level of sports jelly beans [32]. One potential limitation in our study was that the cyclists did not practice the 75-km cycling time trial in the laboratory prior to data collection.

In previous studies, our research group has shown that CHO compared to placebo ingestion during prolonged and heavy exertion attenuates exercise-induced increases in plasma inflammation measures and granulocyte phagocytosis, in part due to higher serum glucose levels and reduced plasma epinephrine and serum cortisol [2]–[5]. Except for IL-10 and IL-8, post-exercise inflammation and phagocytosis, and serum glucose measures were similar between CHO and BAN, and below levels previously reported by our research group for water placebo [2]–[5]. IL-10 exerts anti-inflammatory influences during exercise, and the chemokine IL-8 is a chemoattractant that helps mediate the inflammatory response. The selective difference between CHO and BAN for IL-10 and IL-8 may indicate a slightly higher overall inflammatory response to exercise under BAN conditions, but levels are still below those measured when only water is ingested during exercise [6].

In conclusion, in this randomized, crossover study, cyclists ingesting BAN or CHO at a rate of 0.2 g/kg carbohydrate every 15 min (and one 0.4 g/kg carbohydrate dose pre-exercise) were able to complete 75-km cycling trials with no differences in performance measures. Changes in blood glucose, inflammation, oxidative stress, and innate immune measures were also comparable between BAN and CHO 75-km cycling trials, and similar to what we have previously reported for carbohydrate-fed athletes [2]. Shifts in serum metabolites following BAN and CHO 75-km cycling time trials were extensive, and indicated a similar pattern of increased liver glutathione production and fuel substrate utilization including glycolysis, lipolysis, and amino acid catabolism. FRAP was higher during BAN compared to CHO, but did not translate to diminished oxidative stress as measured with F_2_-isoprostanes. Serum levels of free dopamine increased in BAN compared to CHO, but concentrations were small with no demonstrable cardiovascular effects. Future studies with banana peel-based supplements will reveal if high oral dopamine intake is advantageous for endurance athletes using similar performance and physiological outcomes. In general, ingestion of bananas before and during prolonged and intensive exercise is an effective strategy, both in terms of fuel substrate utilization and cost, for supporting performance.
